# Application of magnetic resonance imaging in transgenic and chemical mouse models of hepatocellular carcinoma

**DOI:** 10.1186/1476-4598-9-94

**Published:** 2010-04-29

**Authors:** Julia Freimuth, Nikolaus Gassler, Nives Moro, Rolf W Günther, Christian Trautwein, Christian Liedtke, Gabriele A Krombach

**Affiliations:** 1 UCSF Helen Diller Family Comprehensive Cancer Center 1450, 3rd Street, San Francisco, CA 94158-9001, USA; 2 Institute of Pathology, University Hospital Aachen, RWTH Aachen University, Pauwelsstrasse 30, D-52074 Aachen, Germany; 3 Department of Medicine III, University Hospital Aachen, RWTH Aachen University, Pauwelsstrasse 30, D-52074 Aachen, Germany; 4 Department of Diagnostic Radiology, University Hospital Aachen, RWTH Aachen University, Pauwelsstrasse 30, D-52074 Aachen, Germany; 5 Department of Radiology, Justus-Liebig University Giessen, Rudolf-Buchheim-Straße 8, D-35392, Giessen, Germany

## Abstract

**Background:**

Hepatocellular carcinoma (HCC) is one of the most common cancers worldwide. The molecular mechanisms underlying hepatocarcinogenesis are still poorly understood. Genetically modified mice are powerful tools to further investigate the mechanisms of HCC development. However, this approach is limited due to the lack of non-invasive detection methods in small rodents. The aim of this study was to establish a protocol for the non-invasive analysis of hepatocarcinogenesis in transgenic mice using a clinical 1.5 Tesla Magnetic Resonance Imaging scanner.

**Results:**

As a model system we used hepatocyte-specific c-myc transgenic mice developing hepatocellular carcinoma at the age of 12-15 months. The scans of the murine livers included axial T2-weighted turbo-spin echo (TSE) images, axial T1-weighted and contrast enhanced T1-weighted gradient echo (fast field echo, FFE) and sagittal true Fast Imaging with Steady state Precession (true-FISP) images. Application of contrast agent was performed via tail vein-catheter and confirmed by evaluation of the altered longitudinal relaxation T1 time before and after application. Through technical adaptation and optimization we could detect murine liver lesions with a minimum diameter of approximately 2 mm and provided histopathological evidence that these MR findings correspond to hepatocellular carcinoma. Tumor growth was repeatedly measured using sequential MRI with intervals of 5 weeks and subsequent volumetric analysis facilitating direct comparison of tumor progression between individual animals. We finally demonstrated that our protocol is also applicable in the widely- used chemical model of N-nitrosodiethylamine-induced hepatocarcinogenesis.

**Conclusion:**

Our protocol allows the non-invasive, early detection of HCC and the subsequent continuous monitoring of liver tumorgenesis in transgenic mice thereby facilitating future investigations of transgenic tumor mouse models of the liver.

## Background

Hepatocellular carcinomas (HCC) represent one of the most common cancers with growing incidence in several regions around the world. The major cause for HCC development is chronic liver disease. Although the exact molecular mechanism leading to HCC progression is still poorly understood, it is well accepted that chronic liver damage is associated with continuous cycles of apoptosis and/or necrosis followed by compensatory proliferation of hepatocytes. This triggers liver fibrosis and cirrhosis but also results in the accumulation of mutations and transformed hepatocytes due to excessive DNA replication and increased mitosis during liver regeneration. Accordingly, a number of genes involved in cell cycle progression and apoptosis including E2F [1,2], c-myc [3] or the pro-apoptotic protease caspase-8 [4] were shown to be deregulated in HCC and the number of identified genes affecting hepatocarcinogenesis is permanently increasing.

During the last decades, the treatment options for patients with advanced HCC have been very limited due to a lack of effective conventional systemic chemotherapy. Promising progress was reported recently by the use of the multi-kinase inhibitor Sorafenib in patients with advanced hepatocellular carcinoma leading to significantly increased overall survival [5]. Consequently, Sorafenib treatment is now the new standard of care for those patients. However, the benefit from Sorafenib treatment concerning overall survival is rather moderate. Therefore, there is still the need for more effective therapies in HCC treatment [6].

In order to define new and better therapeutical targets, strong efforts have been made during the last years to better understand the molecular mechanisms of hepatocarcinogenesis. One promising approach is the analysis of transgenic - or knockout mice specifically targeting tumor promoters or tumor suppressors such as c-myc, p53, p21 and others [7-11]. Indeed, earlier analysis of these animals gave a deep insight into the mechanisms of tumor development in the liver and other organs [8,12,13]. However, recent approaches to measure and compare the tumor incidence and tumor progression between different transgenic mouse lines are still problematic due to a lack of appropriate techniques and detection methods to monitor tumor initiation and tumor growth *in vivo*. Thus, current reports still rely on a huge amount of animals per group which have to be sacrificed at distinct time points for the measurement of tumor size. Subsequently, methods for the continuous monitoring of liver tumor growth in individual transgenic mice need to be developed.

Magnetic Resonance Imaging (MRI) has been used in many clinical, biomedical, chemical and engineering applications. In clinical practice, MRI is used to visualize the internal structure of organs and tissue of the body with a special focus on neurological (brain), cardiovascular and oncological imaging including HCC in particular [14].

In comparison to ultrasound and Computed tomography (CT), MRI has proven to be more reliable when focal liver lesions have to be detected and characterized [15,16]. This greater potential is based on a superior amount of diagnostic information by the assessment of T1- and T2-weighted sequences and the possibility of multiple sequences after injection of contrast media. Application of contrast media leads to its accumulation within the lesions early after injection due to the higher distribution volume and causes a decrease of the T1 relaxation time. Furthermore, an increase in focal liver lesion detection has been reported with the introduction of the liver-specific contrast agent gadoxetic acid disodium [17]. This contrast medium is taken up by intact hepatocytes in the later phases after injection and washed out from hepatocellular carcinoma.

CT can also be used for the detection and characterisation of focal liver lesions. However, this modality exposes small rodents to relatively high doses of radiation therefore potentially influencing hepatocarcinogenesis. Ultrasound on the other hand may not allow objective imaging of all anatomical regions in mice. Based on these considerations, MRI was preferred for longitudinal follow up imaging in rodents. MRI is not associated with radiation exposure and can be repeatedly used for imaging of small animals without any long term effects or alteration of the course of the disease.

Therefore the aim of this study was to establish a protocol for the non-invasive and reliable detection of murine HCC - induction and progression using MRI techniques. As specialized MR scanner for small rodents with higher magnetic field strength are expensive and not available in most clinical research centres, an additional requirement for this protocol was the applicability for regular clinical 1.5 Tesla MR-scanning systems.

## Results

### Establishment of a standard protocol for the MRI analysis of transgenic mice

After correct positioning of the mouse in the MRI scanner in the supine position, a short localizer sequence (see Table 1, row 1) was performed in order to assess effectively the following sequences. Based on these images, axial T1-weighted and T2-weighted scans were planned (see Table 1, row 2 and 3).

**Table 1:** MRI parameters

***No***.	*Sequence type*	*Plane*	*TR/TE*	*Thickness (mm)*	*FOV (mm)*	*Flip angle (degrees)*	*Time*
1	FI2 MST	axial, sagittal, parallel	9.40/4.20	3.00	110.0	30	1:28.5
2	T2TSE	axial	1800/110	2.00	60.0	90	2:34.8
3	T1WFFE	axial	253/6.7	2.00	45.0	80	4:58.3
4	Look-Locker	axial	9.7/4.5	3.00	70.0	15	2:48.0
5	true-FISP	sagittal	9.40/4.20	1.50	80.0	30	1:03.2

The repetition time (TR) parameter determines the user defined repetition time in spin echo sequences while the time of echo (TE) determines the excitation pulse (90°) of the same slice. FOV (*Field of view*) specifies the area to be imaged. Flip determines the angle by which the magnetization vector is rotated around to an axis perpendicular to the main magnetic field during the radio frequency excitation pulse. It can be adjusted for an optimum contrast and/or signal-to-noise ratio.

For better diagnosis and localization of potential liver lesions, a sagittal 3D true-FISP sequence and an axial Look-Locker sequence (see Table 1, row 4 and 5) were performed additionally. The latter one was used to prove successful administration of contrast agent. The acquisition of all pre-contrast scans lasted 13 minutes. After all pre-contrast scans had been performed the contrast medium was administered for optimized lesion detection and the Look-Locker sequences as well as the T1-weighted gradient echo sequences were repeated for analysis.

**Figure 1:**
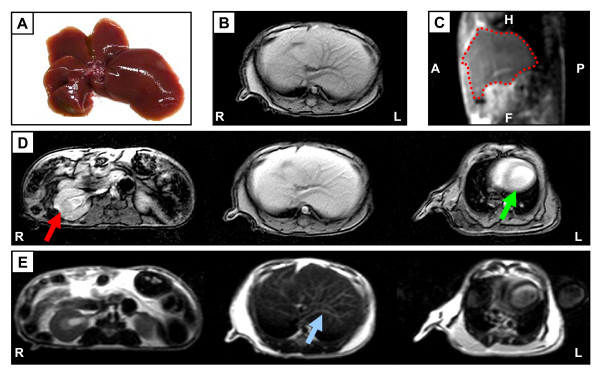
**MR images of a healthy liver from a 35 weeks old WT mouse using a 1.5 Tesla scanner**. (A) Healthy liver of a 35 weeks old mouse after resection. (B) T1-weighted gradient echo image of a healthy mouse liver. (C) Sagittal true-FISP images of the same mouse. The location of the liver is highlighted in red. (D) Distinct images of T1-weighted sequence after application of contrast agent. Structures of different organs are clearly visible and highlighted (red arrow: right kidney; green arrow: heart). (E) Distinct images of healthy mouse liver using T2-weighted sequence, specifically sensitive to water content. Blue arrow pointing to mesh-work of blood vessels. Orientation labeling: R: right; L: left; H: head; A: anterior; P: posterior; F: foot.

The scan parameters were optimized, using wildtype (WT) mice (Figure 1A) by modifying TR/TE values, flip angle and slice thickness (compare Table 1) until the internal liver architecture including the liver vascular system and the gall bladder could clearly be distinguished. In T1-weighted sequences, fat and fat containing tissue (e.g. bone marrow) appear bright (hyperintense) and the structure of organs is clearly detectable. Therefore, in clinical use, T1-scans serve to demonstrate anatomical organ structure. Figure 1B shows a T1-weighted scan of an un-enhanced murine liver, which is characterized by homogeneous signal intensity from the entire liver parenchyma indicating healthy liver tissue. For better localization of the liver and potential pathological lesions, we further performed sagittal true-FISP imaging (Figure 1C), showing the complete liver within the whole body context. The optimized standard protocol for MRI in mice included the use of contrast enhancing sequences. Therefore, a T1-weighted scan was acquired before and after administration of contrast agent allowing better determination of pathological alterations within the tissue. For this purpose, gadoxetic acid disodium was used, which is a gadolinium-based hepatocyte-specific contrast agent for T1-weighted liver imaging. Uptake of gadoxetic acid disodium led to marked signal enhancement in hepatic tissue resulting in a brighter appearance of the liver (compare Figure 1D, medium panel and Figure 1B). Moreover, application of T1-weighted scans allowed a precise display of the anatomical organ structure in mice (Figure 1D) clearly showing defined structure of right kidney, liver and beginning section of the murine heart.

In T2-weighted spin echo sequences water- and fluid-containing tissues (e.g. vessels) are strongly demarcated from the surrounding tissue and have a bright appearance. T2-weighted imaging of a healthy murine liver clearly demonstrated the dark healthy liver parenchyma crossed by a hyperintense mesh-work of connective blood vessels (Figure 1E).

In summary, these initial experiments demonstrated that the optimized parameters shown in Tab. 1 allow the precise imaging of a murine liver in axial and sagittal orientation.

### MRI of tumor-susceptible c-myc transgenic mice

**Figure 2:**
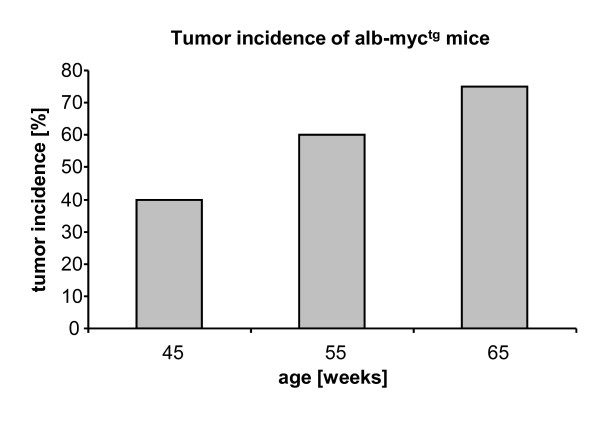
**Tumor incidence of hepatocyte-specific c-myc transgenic mice at the age of 45-65 weeks**. Tumor incidence in alb-myc^tg ^mice used in this study. A subset of animals was sacrificed at the age of 45 weeks (alb-myc^tg^: n = 20), 55 weeks (alb-myc^tg^: n = 10) and 65 weeks (alb-myc^tg^: n = 16), respectively. The extracted livers were analyzed for macroscopically visible tumor nodes. Tumor-positive mice were defined as animals with a minimum of 1 tumor node with a diameter ≥ 1 mm.

Hepatocyte-specific, c-myc transgenic mice (alb-myc^tg^) are a well established animal model for hepatocarcinogenesis. These animals develop slow growing liver tumors similar to a group of human HCCs with better prognosis and survival [3]. As a starting point of our study we determined the age-dependent tumor incidence of alb-myc^tg ^mice showing less than 40% of HCCs in 45 weeks old animals, whereas 80% of the 65 weeks old mice had a minimum of one liver tumor (Figure 2) which is comparable to earlier findings [18].

**Figure 3:**
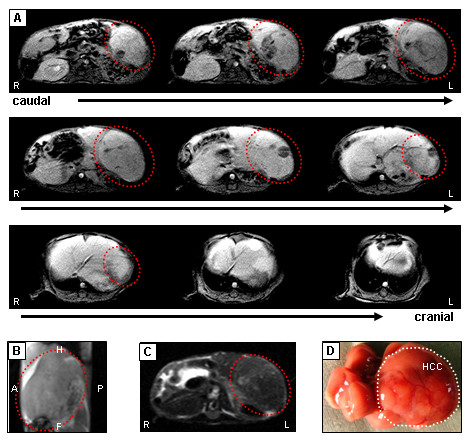
**Detection of advanced HCC in mice**. (A) After *i.v*. injection of the extracellular contrast agent gadoxetic acid disodium, irregular structure of tumor becomes obvious in T1-weighted scans and is highlighted in red. The carcinoma is visible as hypointense signal spread over the organ, due to lack of accumulation of the contrast medium. The surrounding hepatocytes have taken up the contrast agent, which is then present in the intracellular space and causes reduction of T1 relaxation time. (B) Sagittal 3D true-FISP clearly shows strong deformation of the liver. Localization of total liver including carcinoma is highlighted in red. (C) Imaging of the same tumor in a T2-weighted scan revealed an isointense area covering the left hepatic lobe. Red dashed line indicates large tumor. (D) Macroscopic appearance of the respective liver after surgical removal confirms large HCC covering the left part of the liver of an alb-myc^tg ^mouse at 65 weeks of age. White dashed line indicates large tumor. Orientation labeling: R: right; L: left; H: head; A: anterior; P: posterior; F: foot.

To proof whether our optimized MRI parameters (compare Tab. 1) were sufficient to detect HCCs, we started our analysis with a group of n = 24 alb-myc^tg ^mice at the age of 65 weeks and a predicted tumor probability of 80%. Following contrast enhanced T1-weighted imaging and comparison with respective T2-weighted scans, in 15 out of 18 animals pathologically conspicuous areas were detected in sections which could clearly be allocated to the liver. A representative example is shown in Figure 3A-D. The T1-weighted contrast-enhanced sequence (Figure 3A) indicates extensive tumor invasion throughout the entire left hepatic parenchyma. The hypervascular nature and the inhomogeneous internal structure of the tumor are important criteria in the differential diagnosis of this liver compared to the even texture of a healthy animal (compare Figure 1D). Furthermore, sagittal 3D true-FISP sequences displayed a strong deformation of the liver (Figure 3B) compared to wildtype mice (see Figure 1C). A representative image of a T2-weighted sequence showed the dark liver parenchyma together with an isointense tumor node of the left hepatic lobe (Figure 3C). Final confirmation of the diagnosed HCC was obtained upon surgical removal and macroscopical analysis of the liver as depicted in Figure 3D. These initial findings indicate clearly that the developed MRI protocol is applicable to display tumor formation in the murine liver.

### Hypointense MR signals in livers of c-myc transgenic mice correspond to highly tumorigenic tissue sections with strong proliferation

Next, we aimed to provide clear evidence that hypointense signals in murine livers correspond to carcinogenic lesions by correlating our results with histological and molecular findings.

**Figure 4:**
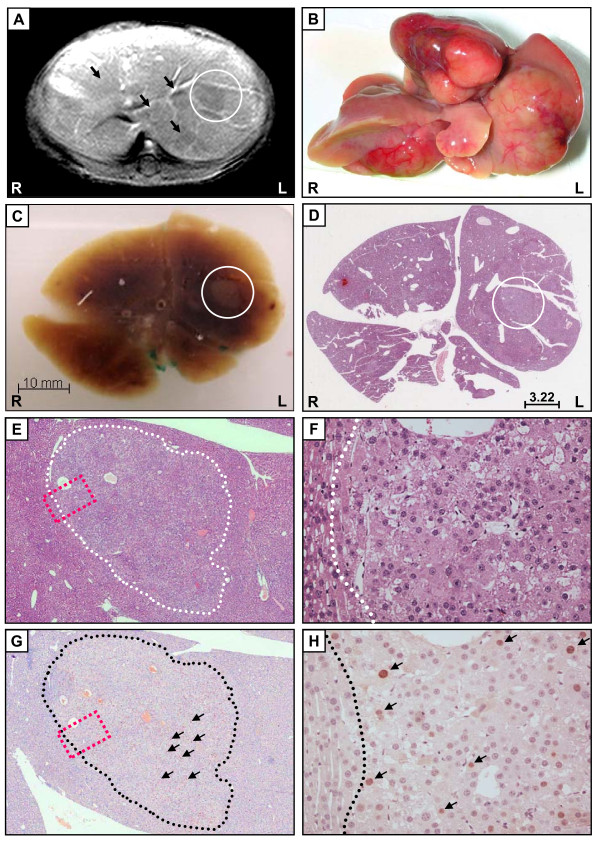
**Hypointense MR signals in livers of alb-myc^tg ^mice correspond to highly tumorigenic tissue sections with strong proliferation**. (A) T1-weighted liver MR image of a 65 weeks old male alb-myc^tg ^mouse after *i.v*. application of gadoxetic acid disodium. Sharply demarcated multiple hypointense foci become obvious within hepatic lobes due to contrast uptake of surrounding hepatic tissue (indicated by arrows); one lesion (white circle) was followed up in subsequent analyses. (B) Macroscopic appearance of explanted liver showing multifocal and sometimes multinodular tumor growth characterized by enhanced vascularisation, discoloration, and destruction of liver segments. (C) Paraffin-embedded section of the complete mouse liver shown in (B). Please note that these serial sections were performed in the same transversal orientation as performed for the MR imaging shown in (A). White circle: macroscopically visible tissue abnormality corresponding to hypointense signal as highlighted in (A). (D) Hematoxylin/eosin staining of the liver section shown in (C). White circle highlights one selected neoplastic lesion surrounded by normal liver tissue. Scale bar indicates diameter of the lesion (mm). (E) Enlarged view (200× original magnification) of mouse liver section with lesions after staining with hematoxylin/eosin. (G) PCNA staining (brown nuclei) of liver tissue as a marker for cell proliferation demonstrates DNA synthesis only in areas identified as HCC and (H) close-up view (200× original magnification) of tumorous areas in PCNA staining. Black arrows indicate PCNA positive nuclei; dashed black line separates healthy liver tissue from tumor nodule; Orientation labeling: R: right; L: left.

65 weeks old animals showing MR signals related to HCC (Figure 4A) were sacrificed and their livers immediately perfused with 4% formaldehyde for *in situ *fixation of the organ. Remarkably, 100% of these MR-positive animals showed macroscopically clear signs of strong HCC development (Figure 4B). Before liver sampling, the orientation of all liver lobes relative to the longitudinal, transversal and sagittal axis of the animals were marked and the respective, complete liver embedded in paraffin used in routine diagnostic procedures.

This approach allowed us to fix the liver in a three-dimensional orientation corresponding to the original *in vivo *orientation. These samples were then sectioned in the axial orientation thus completely comparable to the MR plane of T1- and T2-weighted sequences. Of notice, these crude paraffin sections already showed strong similarity with the MR imaging results as hypointense signals were completely congruent with striking tissue abnormalities in the paraffin sections (compare Figure 4A and 4C). Hematoxylin/eosin staining of whole liver sections confirmed that hypointense MR signals correspond to a pathological liver architecture (Figure 4D) and direct comparison of MR images and histologies revealed that the MRI was able to detect internal liver tumor nodes beginning with diameters of 2 mm (compare 4A and 4D). Detailed histological analysis of the tumor-transformed liver tissue revealed characteristics of hepatocellular neoplasia with some features of hepatocellular carcinoma (Figure 4E-F). Histomorphological stigmata for neoplastic growth of hepatocytes were nodular arrangement of cells variable in size and nuclear configuration, a macrotrabecular pattern of tumor cells, increased mitotic activity, atypical mitosis, destruction of both portal areas and liver lobules, and, occasionally, starting of vascular infiltration with thinning of the vascular vessel wall.

PCNA is an essential factor for initiating DNA synthesis and thus an established marker for proliferating cells. To definitely prove that positive signals identified by MRI correspond to hyperproliferative, tumorigenic nodules, whole liver sections were stained for nuclear PCNA expression. Strong accumulation of PCNA-positive cells was evident exclusively in areas corresponding to liver regions with hypointense signals in T1-weighted MR images (Figure 4G-H) demonstrating that MRI of the murine liver can specifically detect dysplastic nodules with strong hepatocyte proliferation.

### Monitoring of HCC progression

**Figure 5:**
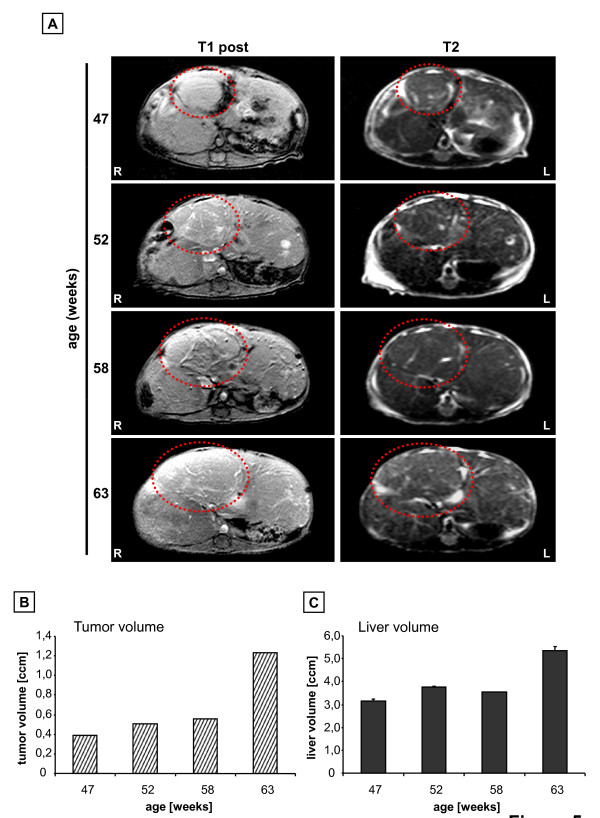
**Monitoring of HCC progression in c-myc^tg ^mice**. (A) Periodical measurement of tumor growth in an alb-myc^tg ^mouse liver at 47, 52, 58 and 63 weeks of age in T1-weighted contrast-enhanced and T2-weighted sequences. Red circle: tumor node measured for volumetric statistics. (B) Chronological progression of tumor growth within 15 weeks demonstrated on one representative tumor node. Volumetric data was obtained using an off-line workstation. (C) Measurement of increasing mouse total liver volume within 15 weeks. Median of three independent measurements is shown. Orientation labeling: R: right; L: left.

A further prerequisite for the application of MRI in murine tumor studies of the liver was the option to monitor tumor growth of HCCs in mice and the respective comparison of tumor size in different animals. Therefore periodical MR imaging was performed in a cohort of alb-myc^tg ^animals starting at the age of around 45 weeks. The animals were monitored every 5 weeks and the tumor growth of detected HCCs was analyzed by measuring liver and tumor volume using the off-line workstation. Tumor measurement was performed on the basis of T1-weighted contrast-enhanced scans. Figure 5A shows a representative example of a T1-weighted contrast-enhanced (left panel) and a T2-weighted sequence (right panel) of a 47 weeks old alb-myc^tg ^mouse with three distinct tumor nodes. Measurement of the median nodule revealed a volume of 0.4 cm^3 ^(Figure 5B). Time-dependent monitoring demonstrated that the size of this nodule increased only slightly up to 58 weeks of age followed by a duplication of tumor size between 58-63 weeks of age (Figure 5B) which corresponded to an increase of total liver volume (Figure 5C). These findings demonstrate that alb-myc^tg ^HCCs are slow progressing tumors as strong tumor progression is restricted to old animals which is in good agreement with earlier reports [3].

Taken together we demonstrated that the protocol established in this study in combination with the available technical equipment allows the measurement and comparison of tumor progression among diverse types of transgenic mice.

### Limitations for detection of small liver tumors in transgenic mice

The last aim of this study was to determine the smallest size of nodules in murine livers, which are already detectable on 1.5 T MRI scans. Therefore, 33 animals at the age of 45-57 weeks (tumor incidence of approximately 50%) were scanned by MRI and checked for hypointense signals in contrast-enhanced T1-weighted images. Immediately after MRI the animals were sacrificed and the respective livers were macroscopically and histologically analyzed for HCCs. The size and location of the macroscopic tumor nodes was measured and correlated to the MRI findings. Overall, 22 out of 33 animals (66%) with evidence of hypointense signals in contrast-enhanced T1-weighted images displayed also tumor nodes with a size between 2 and 10 mm of diameter.

**Figure 6:**
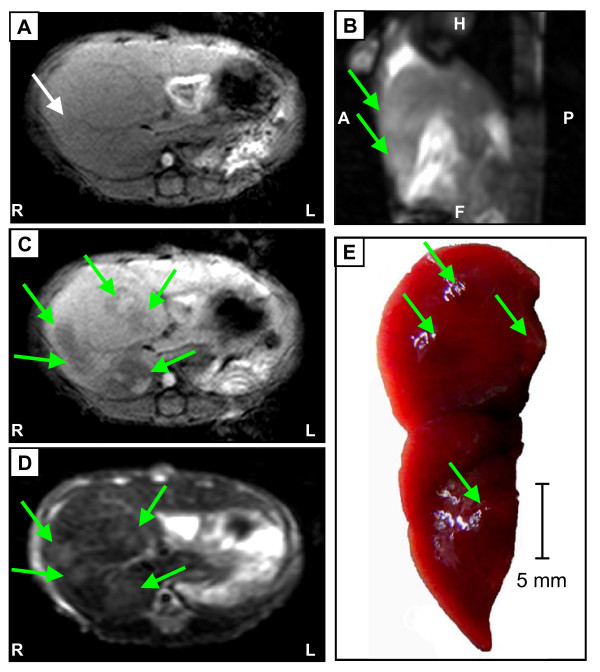
**Detection limits of HCC nodules in an alb-myc^tg ^mice**. (A) In the un-enhanced T1-weighted gradient echo image of murine liver lesions are either not detectable or can be identified only faintly (white arrow). (B) Sagittal true-FISP image demonstrates slightly hyperintense lesion signals (green arrows). (C) In the contrast-enhanced T1 scan of the right hepatic lobes, multiple slightly hypointense lesions are visible (green arrow). These lesions cannot be clearly identified on the pre-contrast scan (A). (D) In the T2-weighted scan hyperintense space-occupying lesions can be seen in the murine liver (green arrows). (E) Surgical situs. View of two hepatic lobes in a sagittal orientation. Visualization of four nodules (green arrows) with 2 mm of diameter within non-tumorgenic liver tissue. Of note, corresponding signals were made visible by 3D true-FISP (compare B), T1-weighted contrast-enhanced (compare C) and T2-weighted (compare D) MR images, but not in un-enhanced T1-weighted image (compare A). Labeling of orientation: R: right; L: left; H: head; A: anterior; P: posterior; F: foot.

The data for one representative animal is shown in Fig 6. Liver lesions could be identified only dimly on T1-weighted scans before contrast-enhancement (Figure 6A) while on the sagittal 3D true-FISP un-enhanced scan (Figure 6B) two hyperintense areas are already visible. However, after application of contrast agent (Figure 6C) multiple hypointense signal patterns appeared in the scan of the right hepatic lobe, which were also detectable in T2-weigthed sequences (Figure 6D). Following resection and macroscopic analysis of the respective liver lobes, the size of macroscopic tumor nodules was measured and correlated to the size of hypointense T1-weighted, contrast-enhanced MRI signals (compare Figure 6C and 6E) by using the off-line workstation. These data revealed that reliable identification of murine tumor nodes beginning at a size of 2 mm in the liver is possible by 1.5 T MRI.

Of note, out of all animals between 45 and 65 weeks of age 100% of tumors with ≥ 4 mm of diameter were verified by MRI, whereas only 70% of tumors with a diameter ≤ 4 mm could be detected with the same settings.

### Application of MRI in an alternative, c-myc-independent mouse model of HCC development

So far, our experiments were exclusively performed in c-myc transgenic mice and wildtype controls. In order to prove that our MR protocol also allows detection of murine HCC in tumor models with different pathogenesis, we decided to use the widely-used, well accepted model of chemically induced HCC in mice using N-nitrosodiethylamine (DEN).

**Figure 7:**
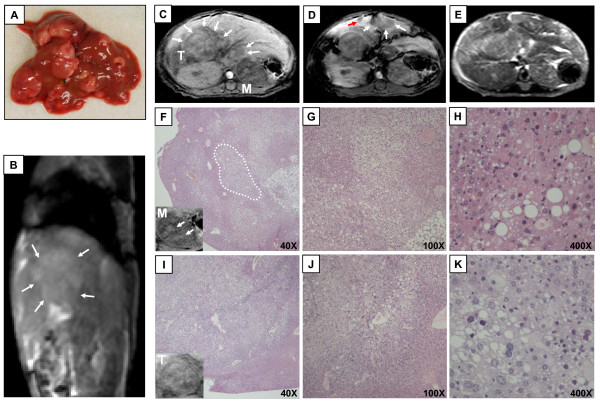
**Application of MRI in the chemical model of N-nitrosodiethylamine induced HCC in mice**. Hepatocarcinogenesis was induced in WT mice by single application of DEN. 45 week old animals were subjected to MRI and subsequently sacrificed. (A) Macroscopic appearance of a representative WT liver 45 weeks after DEN treatment. (B) Sagittal true-FISP imaging displays destruction of normal liver architecture; well defined, slightly hyperintense areas (white arrows) indicate large tumor nodules. (C) Un-enhanced T1-weighted gradient echo image of the same DEN-treated liver. Especially large tumors but also a sub-group of small liver lesions are detectable and appear as hypointense structures (white arrows). (D) Contrast-enhanced T1 scans of the same liver. A sub-group of tumors (arrows) is only detectable after application of contrast agent (gadoxetic acid disodium) and appears highly hypointense. (E) T2-weighted scan showing strong alteration of liver anatomy due to multinodular carcinogenesis. (F-K) Histopathological analysis of two representative tumor nodules. (F) Liver section of one extracted tumor (overview). One example of a nodule-in-nodule like structure is highlighted (white circle) and correlates with structures seen in un-enhanced T1-weighted scans showing hypointense large tumor and embedded hyperintense areas (lower left). (G-H) Enlarged sectional views of (F). (I-K) Histology of a second isolated tumor with moderate differentiation. (I) Section overview, the corresponding appearance of the resected tumor in the MR is shown in the lower left of the figure.(J, K) Enlarged sectional view of (I) showing moderate pleomorphism of tumor cell nuclei and cytoplasm indicating for a moderate differentiation.

The carcinogenic effect of DEN is mediated via its capability of alkylating DNA structures. In most mouse strains, a single injection of DEN in juvenile mice at the age of 14 days leads to multinodular hepatocarcinogenesis within 40-50 weeks in 80-100% of the animals [19]. Genetic analyses revealed that this model well reflects human HCC associated with poor prognosis [3]. We therefore treated a cohort of WT mice with DEN and subjected these animals to MRI 45 weeks after tumor induction. Immediately after imaging the mice were sacrificed and subjected to macroscopic and histopathological analysis of whole liver and tissue sections, respectively. Macroscopic analysis of extracted livers confirmed the multinodular character of this tumor model showing an average of 74 (SD ± 8.7) tumor nodes per liver with a high variation of tumor size ranging from 1-13 mm in size (median tumor size 2.9 ± 2.4 mm Ø, Figure 7A). In the sagittal 3D true-FISP sequences multiple tumors were displayed as clearly defined hyperintense structures with deformation of the normal liver architecture (Figure 7B). In T1-weighted imaging without contrast enhancement (Figure 7C) the sub-group of large tumors could be well displayed as either hypointense- or isointense structures. Of notice, also nodule-within-nodule like structures, which have been suggested as morphologic marker of de-differentiation [20] could be visualized (compare Figure 7F). However, only very few small sized tumors could be verified in the un-enhanced T1-weighted scans. Following contrast enhanced T1-weighted imaging (Figure 7D), a significant number of additional tumors could be displayed as mostly hypointense structures comparable to our results in c-myc transgenic mice. In T2-weighted spin echo sequences the destruction of normal liver morhology was clearly visible (Figure 7E). Moreover, T2-weighted imaging allowed at least the detection of a subgroup of HCC representing the larger tumor nodes.

The histopathological analysis (Figure 7F-K) confirmed our findings and identified the hypointense MR structures as well-differentiated or moderately differentiated nodular-type hepatocellular carcinomas, respectively. The latter ones were morphologically characterized by a more irregular configuration of nuclei and cytoplasm.

In summary, these experiments demonstrated that our MRI protocol for HCC imaging in mice is not restricted to c-myc transgenic animals, but also applicable to chemical HCC models with completely different pathogenesis.

## Discussion

### Rationale for this study

Imaging of small animals on a clinical scanner in this study was challenging, given the small body volume of the rodents. The application of a dedicated small coil allowed for acquisition of images with a high signal/noise ratio, so that spatial resolution was adapted to small sized animals. Another important issue was the development of a stable logistic protocol, including optimized anaesthesia and positioning of the animals for imaging. Sub-optimal anaesthesia allowed the movement of the animal during image acquisition leading to imaging artefacts. This was especially problematic for T1-weighted images acquired shortly after injection of the contrast medium, since this sequence could not be repeated in the same imaging session, due to the already injected dose. Overall, the standardization and streamlining of the logistics was an important prerequisite for acquisition of high quality images in this study.

### Benefit of small rodent studies for improvement of HCC imaging techniques in human patients

Although the major aim of this study was the development of a non-invasive imaging technique for murine HCCs which can be used for further basic research in mice, our results could also potentially contribute to improvement of MR imaging of human hepatocellular carcinoma. In the clinical situation the analysis of MR images with respect to HCC detection mostly depends on the experience of the radiologist as matching of MR results with liver histology is restricted to patients with subsequent liver resection or biopsy. In contrast, MR imaging in mice allows the immediate comparison with liver histology in large cohorts. Moreover, multiple genetic and chemical mouse models are available reflecting the pathogenesis of human hepatocarcinogenesis including steatosis-, fibrosis- and cirrhosis models [21,22] thus defining experimental mouse models as a new attractive tool for the improvement of MR guided liver imaging techniques.

### Limitations of MRI for the detection of murine HCC

Although several studies have demonstrated that MRI represents one of the highest imaging accuracy of all imaging methods available to date [23], the diagnosis of small HCC nodules in human patients by MRI is still challenging. For human HCC >2 cm, MRI showed a detection rate of 100%; however, the detection rate of HCC <1 cm is only 34% [24]. In this context we demonstrated that the monitoring of small single HCC nodules with a diameter of 2 mm is basically feasible in mice. To determine the limit of detection concerning tumor size, a large number of animals with expected onset of tumor development between 45 and 57 weeks of age were screened by MRI and subsequent macroscopic liver analysis. These experiments demonstrated that MR imaging in combination with our optimized parameters detects 100% of murine HCC ≥ 4 mm. However, the detection rate of small nodules (≤ 4 mm) which is important for determining tumor initiation was only 70%. Thus, it is very likely, that nodules ≤ 2 mm will be only partially detected. Nonetheless, in our study 90% of all tumors could clearly be identified by MRI, and these findings were confirmed by macroscopic and histopathologic analysis.

The majority of experiments performed in this study were carried out in c-myc transgenic animals as this is an established and well characterized model of hepatocarcinogenesis. Extensive DNA-profiling analysis revealed that this model corresponds to slow growing human HCC associated with better survival [3,25]. However, to proof that our approach to monitor murine HCC by MRI is not restricted to c-myc dependent hepatocarcinogenesis, we also included a c-myc independent, non-transgenic HCC model in our studies by using the carcinogen N-nitrosodiethylamine (DEN). This model usually generates multinodular HCC within 40 weeks which share a gene expression profile of human HCC with poor prognosis [3,25]. Recently, the DEN model was also used in combination with genetic knockouts of potential tumor promoters [26,27] to further investigate the molecular mechanisms of HCC development.

Our own analysis revealed, that during the early phase of DEN-mediated tumor initiation (24 weeks after DEN-induction), MRI was not efficient in detecting small tumor nodules in the liver (data not shown). This might be due to the nature of these tumors with small, multinodular appearance with the challenge to distinguish between single lesions. These problems are enhanced by the small size of the analyzed object (mice versus humans) and a relatively low signal/noise ratio due to the magnetic field strength of 1.5T of the used MR scanner thus defining some limitations of clinical MR scanners in monitoring murine HCC nodules. However, such difficulties might also have been observed in larger subjects, since the multiple small tumours have a low contrast compared to the surrounding tissue. Nevertheless, we clearly could detect advanced, DEN-induced liver tumors in mice 45 weeks after tumor induction, which were retrospectively characterized as well- or moderately differentiated HCC, further demonstrating that clinical 1.5T MR scanners are appropriate tools for liver cancer research in mice.

It was shown before that it is basically possible to detect hepatocellular carcinoma in rodents with a clinical 1.5T MR scanner e.g. by using an orthotopic tumor cell implantation model in rats [28]. Nevertheless, our own study implicates significant methodical progress in comparison to these studies for several reasons: 1.5T MR imaging in rats is less challenging due to their significantly larger body volume compared to mice resulting in a better signal/noise ratio. Moreover, mechanistical studies on hepatocarcinogenesis in rats are restricted to models of chemical tumor induction or tumor cell transplantation as gene knockout technologies in rats are currently not established and appropriate transgenic rat models are limited. Although orthotopic implantation of genetically modified HCC cells in the liver and subsequent MR imaging of tumor progression- or regression is a powerful tool e.g. for drug screenings, the majority of future animal experiments concerning HCC research will be definitely performed in genetic modified knock out mice targeting oncogenes or tumor suppressors. Optimizing MR imaging of liver tumors in mice will further support this approach.

Only a few studies have described the monitoring of murine HCC by MRI so far and these studies mainly took advantage of MR scanners specialized for small animals with a magnetic field strength of 4.7 T or higher [29]. However, these scanners are expensive and the availability is restricted.

## Conclusion

In summary, we demonstrated that detection of murine HCC initiation and progression in transgenic mouse models is feasible with clinical 1.5 T MR scanners thus allowing further analysis of potential oncogenes and tumor suppressors in the liver by comparison of respective tumor-susceptible transgenic mouse models. Nevertheless, limitations in reliable detection and characterization of small HCC remain an area of further investigation. This will help to gain better insight and to understand the underlying molecular mechanisms of HCC initiation and development.

## Materials and methods

### Housing and breeding of mice

All animals used for experiments were maintained in a temperature-controlled room with 12-hour light/dark cycle. Animal experiments were approved by the authority for environment conservation and consumer protection of the state North Rhine-Westfalia (LANUV, Recklinghausen, Germany; reference number AZ 50.203.2-AC 5a,39/05) and the University Hospital Aachen Animal Care Facility's guidelines. For our study we used transgenic mice carrying a c-myc transgene under the control of the hepatocyte-specific albumin promoter (alb-myc^tg^) of male gender as previously described [18]. As controls, we used wildtype (WT) littermates derived from heterozygous breeding couples.

### Chemical induction of hepatocellular carcinoma in mice using N-nitrosodiethylamine (DEN)

For chemical induction of hepatocellular carcinoma, 14-day old wildtype mice on a mixed C57BL6/129ola background were injected once with 25 mg/kg body weight DEN (Sigma) intraperitoneally as described earlier [30,31]. Usually, these mice developed multinodular HCC between 24 - and 40 weeks of age.

### Experimental protocol for MRI analysis in mice

All animal interventions including MRI analysis in mice were carried out under anaesthesia using 100 mg/kg Ketamin and 10 mg/kg Rompun (*i.p*.). For contrast enhancement 0.025 mmol/kg body weight gadoxetic acid disodium (Gd-EOB-DTPA, Primovist^®^, Schering, Berlin, Germany) was administered by tail vein injection using a catheter with injection port (Biovalve^® ^22G; 1,0 × 25 mm, Vygon, Ecouen, France).

All MR measurements were performed using an Achieva 1.5-Tesla Scanner MR System (Philips Medical Systems, Best, The Netherlands). A 47 mm diameter coil (Philips Medical Systems) was used for signal reception. For volumetric analysis we used an off-line workstation (View Forum, Philips Medical Systems).

### Extraction and sectioning of complete livers

For preparation of whole livers, mice were anesthetized and livers were immediately perfused through the inferior vena cava with 4% formaldehyde/PBS for 20 minutes. Subsequently the complete liver was extracted and fixed in 4% formaldehyde/PBS for 24-48 hours and then embedded in paraffin (Shandon Histoplast paraffin, Thermo Scientific, Egelsbach, Germany) according to its original three-dimensional topography. In order to perform tissue sections from formalin-fixed and paraffin-embedded liver which exactly parallels MR images, the surface of the formalin-fixed liver was marked with a color code corresponding to topographical hallmarks (i.e. left, right, cranial, caudal). Afterwards the whole organ was embedded in paraffin and orientated as found in the MR images. Serial 4 μm sections were performed and subsequently stained with hematoxylin/eosin following routine protocols.

### Immunhistochemistry

PCNA immunhistochemistry was performed on 5 μm paraffin embedded sections with fixation in 4% formaldehyde/PBS for 5 min using an anti-PCNA antibody (Dianova, Hamburg, Germany) at a dilution of 1:200 and a secondary goat anti-mouse-HRP antibody (Santa Cruz Biotechnology, Santa Cruz, CA) at a dilution of 1:100. Final staining was performed with DAB solution (Sigma-Aldrich, Taufkirchen, Germany) and counterstaining with hematoxylin.

## Abbreviations

HCC: hepatocellular carcinoma; MRI: magnetic resonance imaging; TSE: turbo spin echo; FFE: fast field echo; true-FISP: true fast imaging in steady state precession; TR: repetition time; TE: time of echo; FOV: field of view; CT: computed tomography; WT: wildtype; PBS: phosphate-buffered saline; PCNA: proliferation cell nuclear antigen.

## Competing interests

The authors declare that they have no competing interests.

## Authors' contributions

JF performed most of the *in vivo *experiments, analyzed data, carried out immunohistochemical staining of tissue slides and contributed to manuscript draft. NG performed histological and pathological analysis of liver samples and contributed to manuscript draft. NM performed the chemical induced hepatocarcinogenesis in wildtype mice and analyzed tumor samples. RWG provided MRI equipment and supervised imaging experiments. CT coordinated the study and contributed to manuscript preparation. CL designed the study and wrote the manuscript. GAK coordinated the experiments, analyzed the MRI data and wrote the manuscript.

All authors read and approved the final manuscript.
